# Decreased monocyte-to-lymphocyte ratio was associated with satisfied outcomes of first-line PD-1 inhibitors plus chemotherapy in stage IIIB-IV non-small cell lung cancer

**DOI:** 10.3389/fimmu.2023.1094378

**Published:** 2023-01-26

**Authors:** Liang Zheng, Anning Xiong, Shuyuan Wang, Jianlin Xu, Yinchen Shen, Runbo Zhong, Jun Lu, Tianqing Chu, Wei Zhang, Ying Li, Xiaoxuan Zheng, Baohui Han, Hua Zhong, Wei Nie, Xueyan Zhang

**Affiliations:** Department of Pulmonary, Shanghai Chest Hospital, School of Medicine, Shanghai Jiao Tong University, Shanghai, China

**Keywords:** non-small cell lung cancer (NSCLC), prognostic value, immune checkpoint inhibitors (ICI), chemotherapy, monocyte-to-lymphocyte ratio (MLR)

## Abstract

**Objectives:**

Immune-checkpoint inhibitors (ICIs) combined with chemotherapy are more widely used than monotherapy and have shown better survival in patients with advanced non-small cell lung cancer (NSCLC) without oncogenic driver alterations. The monocyte-to-lymphocyte ratio (MLR) might predict the treatment outcomes of ICI therapy in advanced NSCLC patients but has not yet been investigated. In addition, the cutoff of MLR is controversial. Therefore, the present study aimed to explore the associations between changes in MLR at the initial stage of treatment and clinical outcomes in stage IIIB-IV NSCLC patients receiving first-line PD-1 inhibitor combined with chemotherapy.

**Methods:**

The present study included 139 stage IIIB-IV NSCLC patients treated with first-line PD-1 inhibitor combined with chemotherapy. The blood results were assessed 10 days before initiation of PD-1 inhibitor-based combination therapy (time point 1, baseline) and before the third cycle of combined therapy (time point 2). Compared to altered MLR, neutrophil-to-lymphocyte ratio (NLR), and platelet-to-lymphocyte ratio (PLR) in baseline and in time point 2, patients were divided into decreased MLR/NLR/PLR and increased MLR/NLR/PLR groups. The objective response rate (ORR), progression-free survival (PFS), and the association with the changes in blood indicators were analyzed.

**Results:**

A total of 48 patients were categorized in the decreased MLR group and 91 in the increased MLR group. Patients with decreased MLR had a significantly higher ORR in the univariate (P<0.001) and multivariate (P<0.001) Cox proportional hazards models. On the other hand, decreased MLR was significantly associated with prolonged PFS in the univariate (P=0.007) and multivariate (P=0.016) analyses. Next, 91 patients comprised the decreased NLR group and 48 as the increased NLR group. Patients with decreased NLR exhibited high ORR (P=0.001) and prolonged PFS in univariate analysis (P=0.033). Then, 64 patients comprised the decreased PLR group and 75 the increased PLR group. Decreased PLR was significantly associated with high ORR in univariate (P<0.001) and multivariate (P=0.017) analyses. The subgroup analyses showed that decreased MLR was significantly associated with satisfactory outcomes in patients with all PD-L1 expressions.

**Conclusion:**

Decreased MLR was associated with high ORR and long PFS and might have a potential predictive value in patients with stage IIIB-IV NSCLC treated with first-line PD-1 inhibitor combined with chemotherapy. In addition, changes in MLR might have predictive value in all PD-L1-expressing populations. Decreased NLR and PLR also showed improved survival, suggesting that changes in NLR and PLR may be complementary to predicting prognosis.

## Introduction

The use of immune-checkpoint inhibitors (ICIs), specifically those targeting PD-1 or its programmed cell death ligand-1 (PD-L1), yields a durable response and prolongs the survival of patients with advanced non-small cell lung cancer (NSCLC) with high PD-L1 expression (1). However, the efficacy of monotherapy in patients is limited (2). Many studies have shown that chemotherapy-immunotherapy combinations achieved prolonged progression-free survival (PFS) and overall survival (OS) in advanced NSCLC patients regardless of the PD-L1 expression level and also improved survival than chemotherapy alone (3). ICIs, in combination with chemotherapy, have gradually become the first-line therapy for advanced NSCLC patients without oncogenic driver alterations (4). However, some patients suffer from treatment resistance, exhibiting severe immune-related adverse events (irAEs) (5). Therefore, finding precise and reliable biomarkers for immunotherapy plus chemotherapy is imperative.

To date, predictive biomarkers for ICIs plus chemotherapy have not been identified. PD-L1 expression and tumor mutation burden (TMB) are candidate biomarkers for ICIs plus chemotherapy (6). PD-L1 on tumor cells has been the predictive biomarker of response to ICIs, however, its predictive value is not accurate as the PD-L1 tumor proportion score (TPS) groups might achieve significant survival benefits, including those with TPS <1% (7). In addition, PD-L1 exhibits temporal changes in the expression and intra-tumoral heterogeneity and can only be tested on tissue specimens *via* an invasive operation (8, 9). The assessment of PD-L1 expression during treatment becomes challenging. Blood-based TMB (bTMB) can be detected from the blood and has several advantages over tissue biopsy, such as easily repeated collection over time. Nonetheless, it also has shortcomings as a marker of therapeutic effects. Currently, no distinct thresholds define high or low bTMB levels. In addition, the correlation between bTMB and prognosis presents a non-linear association, and establishing a cutoff becomes challenging (10, 11). Therefore, it lacks effective and convenient markers for immunotherapy plus chemotherapy.

Inflammation can affect disease progression and the survival of many cancers (12). Over the last few years, the prognostic and predictive value of inflammatory-related peripheral blood biomarkers in NSCLC patients receiving immunotherapy has been investigated in-depth. Several studies have shown that high neutrophil-to-lymphocyte ratio (NLR) and platelet-to-lymphocyte ratio (PLR) are prognostic markers associated with poor survival in advanced NSCLC patients with immunotherapy (13, 14). However, these studies mainly explored the cutoff, but the inflammatory markers are not linearly related to patient outcomes (15). Therefore, the cutoff of the selected inflammatory markers was uncertain and controversial. Moreover, inflammatory cells, including monocytes and lymphocytes, are commonly associated with tumor prognosis. Monocytes affect the tumor microenvironment by inducing angiogenesis and immune tolerance, and the spread of tumor cells and monocytes has a stronger phagocytosis compared to other blood cells (16). A decrease in the level of lymphocytes induces the release of several suppressive immunological mediators (17). Previous studies have shown that a decrease in the monocyte-to-lymphocyte ratio (MLR) is significantly associated with the good effects of nivolumab monotherapy (18). These findings suggested that MLR is a promising biomarker to predict the survival benefit of ICIs in advanced NSCLC patients. However, the MLR has predictive value for chemotherapy-immunotherapy combinations in advanced NSCLC patients without sensitive driver mutations, but has not yet been investigated.

MLR, NLR, and PLR are readily available inflammatory biomarkers, and the test is clinically convenient and practically noninvasive. We can also monitor blood parameters dynamically for a prolonged duration. However, it is difficult to determine cutoff values for these inflammatory markers. Therefore, the present study aimed to investigate the predictive role of changes in MLR at the initial stage of treatment in stage IIIB-IV NSCLC patients treated with first-line PD-1 inhibitor plus chemotherapy. In addition, whether changes in NLR and PLR can predict the prognosis of patients was investigated.

## Materials and methods

### Patients

**Figure 1:**
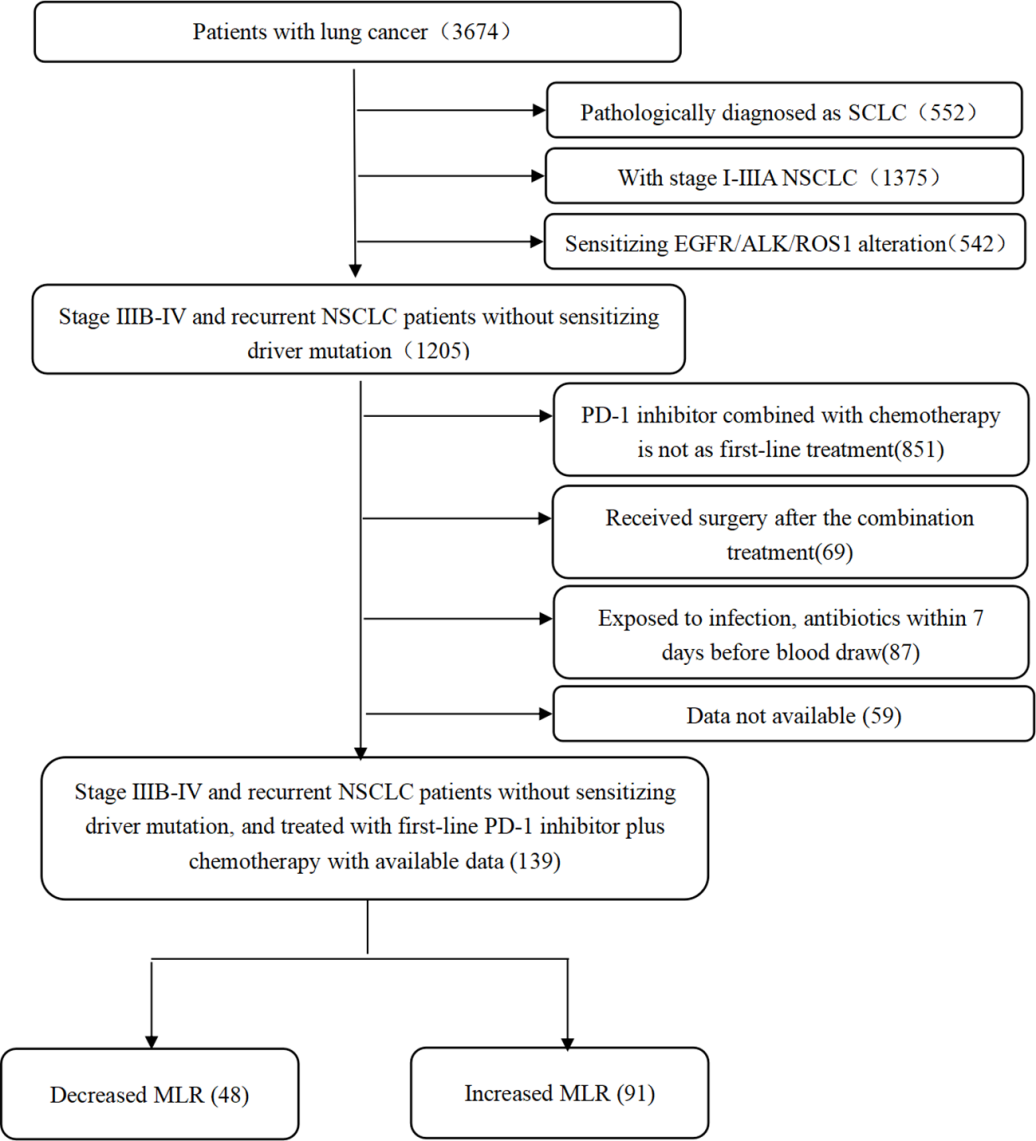
Patient disposition. NSCLC, non-small cell lung cancer; MLR, monocyte-to-lymphocyte ratio.

We retrospectively identified and included 139 stage IIIB-IV NSCLC patients treated with a first-line PD-1 inhibitor (pembrolizumab, sintilimab, or tislelizumab) combined with chemotherapy at Shanghai Chest Hospital, from January 2019 to June 2021. The inclusion criteria for patients were as follows: (1) histopathological confirmation of NSCLC; (2) initial stage IIIB-IV or recurrence after curative surgery; (3) receiving first-line PD-1 inhibitor treatment combined with chemotherapy. The exclusion criteria were as follows: (1) sensitized EGFR/ALK/ROS1 alteration; (2) underwent surgery after the combination treatment; (3) exposed to infection and antibiotics within 7 days before blood draw; (4) data not available (**Figure 1**).

The study was approved by the ethics review board at Shanghai Chest Hospital (Shanghai, China). All participants provided written informed consent.

### Treatment and data collection

Patients were assessed every 2-3 months by computed tomography (CT) scan. The clinical features and laboratory parameters of the patients, including age, gender, smoking history, tumor histology, TNM staging, and PD-L1 expression, were obtained from medical records. Also, the blood results and the incidence of irAEs were recorded. The patients were followed up regularly during the treatment.

Patients received the following therapy: 200 mg pembrolizumab, 200 mg sintilimab, or 200 mg tislelizumab intravenously every 3 weeks. The treatment was continued until tumor progression, development of unacceptable drug toxicity, or death. The combination chemotherapy was platinum-based doublet chemotherapy, while the other drugs, including pemetrexed, docetaxel, paclitaxel/nab-paclitaxel, paclitaxel liposomes, vinorelbine, and gemcitabine were administered according to tumor histology. Peripheral blood samples were collected 10 days before initiation of PD-1 inhibitor-based combination therapy (time point 1, baseline) and before the third cycle of combined therapy (time point 2). If the disease progressed early, before the expected time point 2, a sample of peripheral blood was collected when assessing the disease progression by CT. Complete blood counts, including absolute monocyte count (AMC), absolute neutrophil count (ANC), absolute lymphocyte count (ALC), and platelet count, were recorded at the baseline (time point 1) and at the third cycle of combined therapy (time point 2) (19).

MLR was defined as the ratio of AMC to ALC, NLR as the ratio of ANC to ALC, and PLR as the ratio of platelet counts to ALC. Inflammatory biomarkers (IBs), including MLR, NLR and PLR, were calculated in two time points. The first was before initiation of PD-1 inhibitor-based combination therapy, during which the blood indicators were known as pre-inflammatory biomarkers (pre-IBs). The other stage was prior to the therapy, termed as post-inflammatory biomarkers (post-IBs). The delta-IBs were calculated as follows: delta-IBs=post-IBs/pre-IBs−1. If delta-IBs were >0, it was defined as an increase, while ≤ 0 was deemed as a decrease.

PD-L1 expression was analyzed using PD-L1 immunohistochemistry (IHC) 22C3 pharmDx (Dako), and positive membranous staining of at least 1% of the tumor cells was defined as positive, while <1% was defined as negative. Next, we subdivided the positive status into the high (≥50%) and low (1–49%) expression categories.

### Statistical analysis

Objective response rate (ORR) was defined as the percentage of patients who achieved a complete response (CR) or partial response (PR) among all the treated patients. PFS was defined as the duration from the receiving first-line PD-1 inhibitor combined with chemotherapy to the date of first documented disease progression or death.

χ^2^ test was used to examine the differences in baseline and patient characteristics between the decreased and increased groups. Kaplan-Meier survival curves were generated and the log-rank test was applied to examine the survival difference between the two groups. Factors associated with ORR were tested with logistic regression in univariate and multivariate analyses. The Cox proportional hazards model was applied to calculate the hazard ratios (HRs) and evaluate the factors independently associated with PFS. Receiver operating characteristic (ROC) curves were used to compare the baseline values and the changes in MLR, NLR, and PLR. SPSS 25.0 software (SPSS, Chicago, IL, USA) and GraphPad Prism software (Prism 8) was used for all the statistical analyses. A two-sided p-value<0.05 was considered statistically significant.

## Results

### Characteristics of patients

A total of 139 stage IIIB-IV NSCLC patients treated with first-line PD-1 inhibitor combined with chemotherapy participated in this study. The baseline characteristics of these patients are summarized in **Table 1**. Most patients were >65-years-old (51.8%), and the male proportion was 79.9%, smokers were 74.8%, and 71.9% were in stage IV or had recurrence after surgery (71.9%). Moreover, 79 (56.8%) patients had non-squamous cell carcinoma (including adenocarcinoma and lymphoepithelioma-like carcinoma), 44 (31.7%) had squamous cell carcinoma, and 16 (11.5%) had NSCLC not otherwise specified. For PD-L1 expression, 35 (25.2%) patients had a TPS of ≥50%, 45 (32.4%) patients had a TPS of 1–49%, 42 (30.2%) patients had a TPS of ≤1%, and 17 (12.2%) patients were yet to be diagnosed.

**Table 1:** Patient characteristics.

Characteristic	Patients
Total number	139
Age (years), n (%)	
<65	67 (48.2)
≥65	72 (51.8)
Gender, n (%)	
Male	111 (79.9)
Female	28 (19.1)
Smoking history	
Never	35 (25.2)
Current/former	104 (74.8)
Histology, n (%)	
Squamous	44 (31.7)
Non-squamous*	79 (56.8)
NOS	16 (11.5)
TNM stage, n (%)	
IIIB-IIIC	39 (28.1)
IV/Recurrence	100 (71.9)
PD-L1 expression, n (%)	
TPS≥50%	35 (25.2)
1%≤TPS ≤ 49%	45 (32.4)
TPS<1%	42 (30.2)
Unknown	17 (12.2)

*Non-squamous tumor included adenocarcinoma, lymphoepithelioma-like carcinoma.

NOS, not otherwise specified; PD-L1, programmed cell death-Ligand 1; TPS, tumor proportion score.

A total of 48 (34.5%) patients had decreased MLR before the third cycle of PD-1 inhibitor-based combination therapy, while 91 (65.5%) had increased MLR. These patients were divided into decreased and increased MLR groups. Similarly, 139 patients were divided into decreased and increased NLR groups and decreased and increased PLR groups. According to **Table 2**, the demographic and clinical characteristics of the patients between the decreased and increased groups did not show any significant differences (P>0.05).

**Table 2:** Correlation between blood parameters and clinicopathological characteristics.

Characteristic	MLR			NLR			PLR		
	Decrease (n=48)	Increase (n=91)	P	Decrease (n=91)	Increase (n=48)	P	Decrease (n=64)	Increase (n=75)	P
Age (years), n (%)									
<65	25 (52.1)	42 (46.2)	0.506	44 (48.4)	23 (47.9)	0.961	34 (53.1)	33 (44.0)	0.283
≥65	23 (47.9)	49 (53.8)		47 (51.6)	25 (52.1)		30 (46.9)	42 (56.0)	
Gender, n (%)									
Male	39 (81.3)	72 (79.1)	0.766	71 (78.0)	40 (83.3)	0.458	54 (84.4)	57 (76.0)	0.220
Female	9 (18.7)	19 (20.9)		20 (22.0)	8 (16.7)		10 (15.6)	18 (24.0)	
Smoking history									
Never	11 (22.9)	24 (26.4)	0.655	26 (28.6)	9 (18.8)	0.205	14 (21.9)	21 (28.0)	0.407
Current/former	37 (77.1)	67 (73.6)		65 (71.4)	39 (81.2)		50 (78.1)	54 (72.0)	
Histology, n (%)									
Squamous	19 (39.6)	25 (27.5)	0.069	26 (28.6)	18 (37.5)	0.298	23 (36.0)	21 (28.0)	0.142
Non-squamous*	21 (43.7)	58 (63.7)		56 (61.5)	23 (47.9)		31 (48.4)	48 (64.0)	
NOS	8 (16.7)	8 (8.8)		9 (9.9)	7 (14.6)		10 (15.6)	6 (8.0)	
TNM stage, n (%)									
IIIB-IIIC	11 (22.9)	28 (30.8)	0.327	21 (23.1)	18 (37.5)	0.072	17 (26.6)	22 (29.3)	0.717
IV/Recurrence	37 (77.1)	63 (69.2)		70 (76.9)	30 (62.5)		47 (73.4)	53 (70.7)	
PD-L1 expression, n (%)									
TPS≥50%	14 (29.2)	21 (23.1)	0.821	27 (29.7)	8 (16.7)	0.264	17 (26.6)	18 (24.0)	0.743
1%≤TPS ≤ 49%	16 (33.3)	29 (31.9)		30 (33.0)	15 (31.2)		23 (35.9)	22 (29.3)	
TPS<1%	13 (27.1)	29 (31.9)		25 (27.5)	17 (35.4)		17 (26.6)	25 (33.3)	
Unknown	5 (10.4)	12 (13.1)		9 (9.8)	8 (16.7)		7 (10.9)	10 (13.4)	
Radiotherapy									
Yes	8 (16.7)	21 (23.1)	0.377	21 (23.1)	8 (16.7)	0.377	13 (20.3)	16 (21.3)	0.883
No	40 (83.3)	70 (76.9)		70 (76.9)	40 (83.3)		51 (79.7)	59 (78.7)	
irAEs									
Yes	13 (27.1)	14 (15.4)	0.097	18 (19.8)	9 (18.8)	0.884	12 (18.7)	15 (20.0)	0.853
No	35 (72.9)	77 (84.6)		73 (80.2)	39 (81.2)		52 (81.3)	60 (80.0)	

*Non-squamous tumor included adenocarcinoma, lymphoepithelioma-like carcinoma.

MLR, monocyte-to-lymphocyte ratio; NLR, neutrophil-to-lymphocyte ratio; PLR, platelet-to-lymphocyte ratio; NOS, not otherwise specified; PD-L1, programmed cell death-Ligand 1; TPS, tumor proportion score; irAEs; immune-related adverse events.

### Analysis for ORR

**Figure 2:**
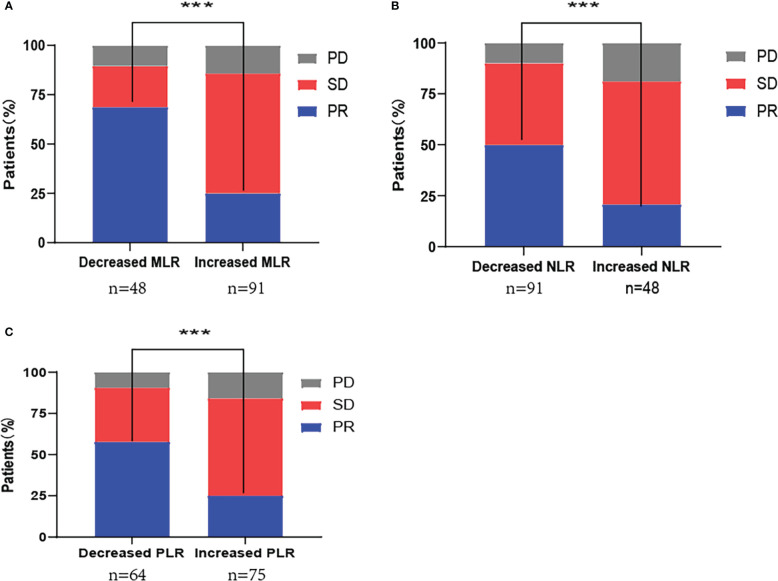
First-line PD-1 inhibitor combination with chemotherapy response distribution by changes in **(A)** monocyte-to-lymphocyte ratio (MLR); **(B)** neutrophil-to-lymphocyte ratio (NLR); **(C)** platelet-to-lymphocyte ratio (PLR). PD, progressive disease; SD, stable disease; PR, partial response; ***P<0.001.

The ORR for patients in the decreased and increased MLR groups was 68.8% and 25.3%, respectively (P<0.001). The ORR for decreased and increased NLR groups was 50.5% and 20.8%, respectively (P<0.001), and that for decreased and increased PLR group patients was 57.8% and 25.3%, respectively (P<0.001) (**Figure 2**).

**Figure 3:**
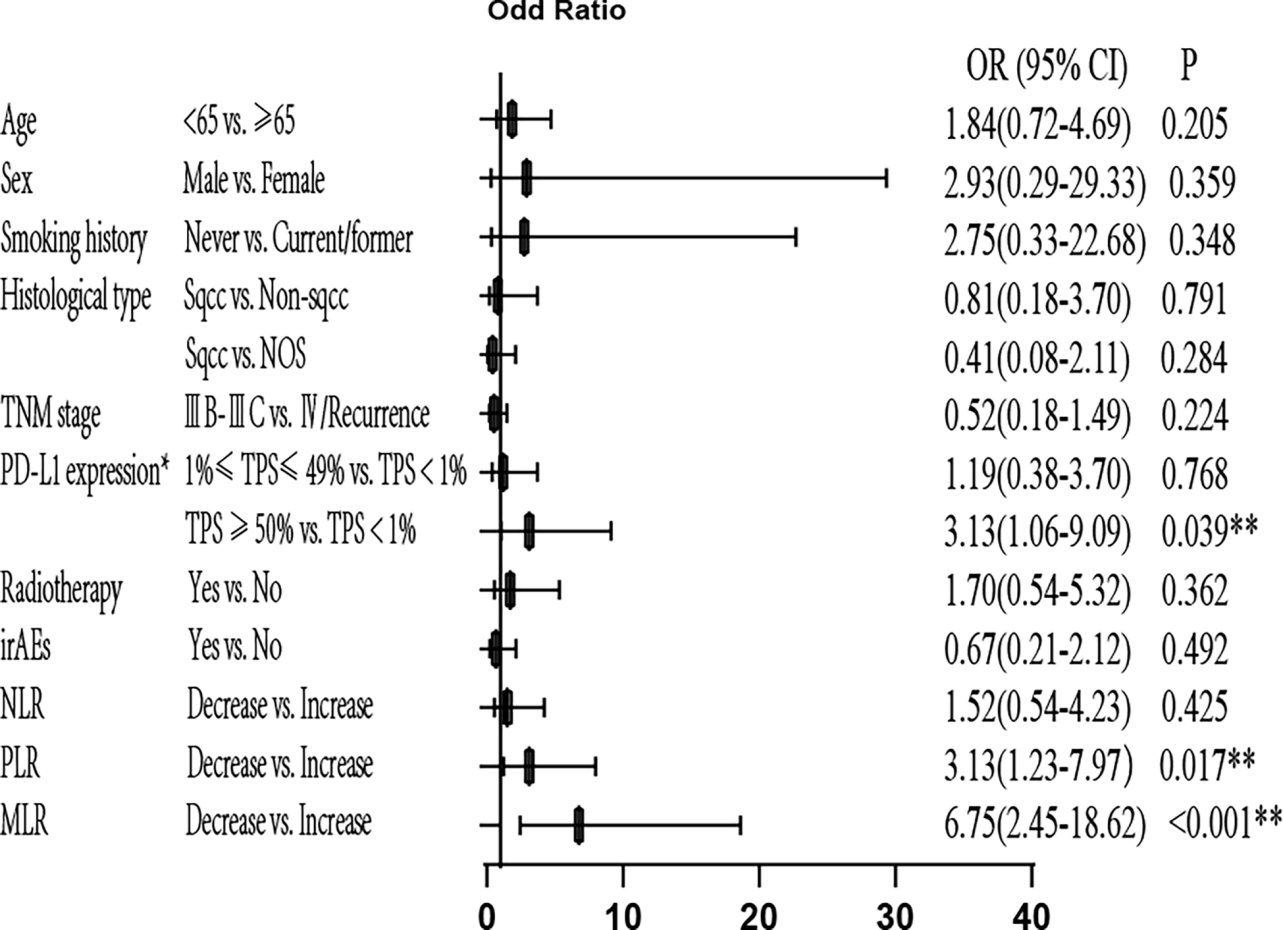
Multivariate analysis of ORR. *Only for patients with available PD-L1 expression data (patients with unknown PD-L1 expression were excluded). ORR, objective response rate; OR, odds ratio; PD-L1, programmed cell death-ligand 1; TPS, tumor proportion score; irAEs, immune-related Adverse Events; NLR, neutrophil-to-lymphocyte ratio; PLR, platelet-to-lymphocyte ratio; MLR, monocyte/lymphocyte ratio; **P<0.05 indicates statistical significance.

Next, we performed univariate and multivariate analyses for ORR and found that age, sex, smoking history, histological type, Tumor Node Metastasis (TNM) stage, radiotherapy, and irAEs had no significant association with ORR (P>0.05). Patients with 1–49% PD-L1 expression showed significantly higher ORR compared to patients with negative PD-L1 expression in univariate analysis [odds ratio (OR)=2.50, 95% confidence interval (CI): 1.04–5.88; P=0.039]. Patients with at least 50% PD-L1 expression showed significantly higher ORR compared to negative PD-L1 expression in multivariate analysis (OR=3.13, 95% CI: 1.06–9.09; P=0.039). Decreased MLR was significantly associated with high ORR in univariate (OR=6.50, 95% CI: 3.01–14.08; P<0.001) and multivariate (OR=6.75, 95% CI: 2.45–18.62; P<0.001) analyses. Decreased NLR was significantly associated with high ORR in univariate (OR=3.88, 95% CI: 1.73–8.72; P=0.001) analysis, but no association was observed with ORR in multivariate analysis (P>0.05). Decreased PLR was significantly associated with high ORR in univariate (OR=4.04, 95% CI: 1.97–8.29; P<0.001) and multivariate (OR=3.13, 95% CI: 1.23–7.97; P=0.017) analyses. Therefore, high PD-L1 expression and decreased MLR and PLR are independently associated with high ORR (**Figure 3** and **Supplementary Figure 1**).

### Analysis for PFS

**Figure 4:**
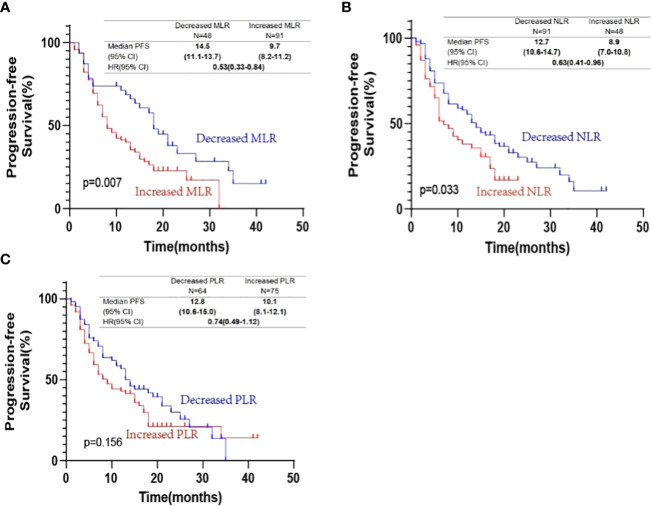
Kaplan-Meier progression-free survival curves according to changes in **(A)** monocyte-to-lymphocyte ratio (MLR); **(B)** neutrophil-to-lymphocyte ratio (NLR); **(C)** platelet-to-lymphocyte ratio (PLR).

The Kaplan-Meier plots in **Figure 4** show a decrease in MLR and NLR at the third cycle of PD-1 inhibitor-based combination therapy from baseline, which was significantly associated with prolonged PFS (MLR: HR, 0.53; 95% CI: 0.33–0.84; P=0.007; NLR: HR, 0.63; 95% CI: 0.41–0.96; P=0.033). However, a decrease in PLR did not show any significant association (P=0.156).

**Figure 5:**
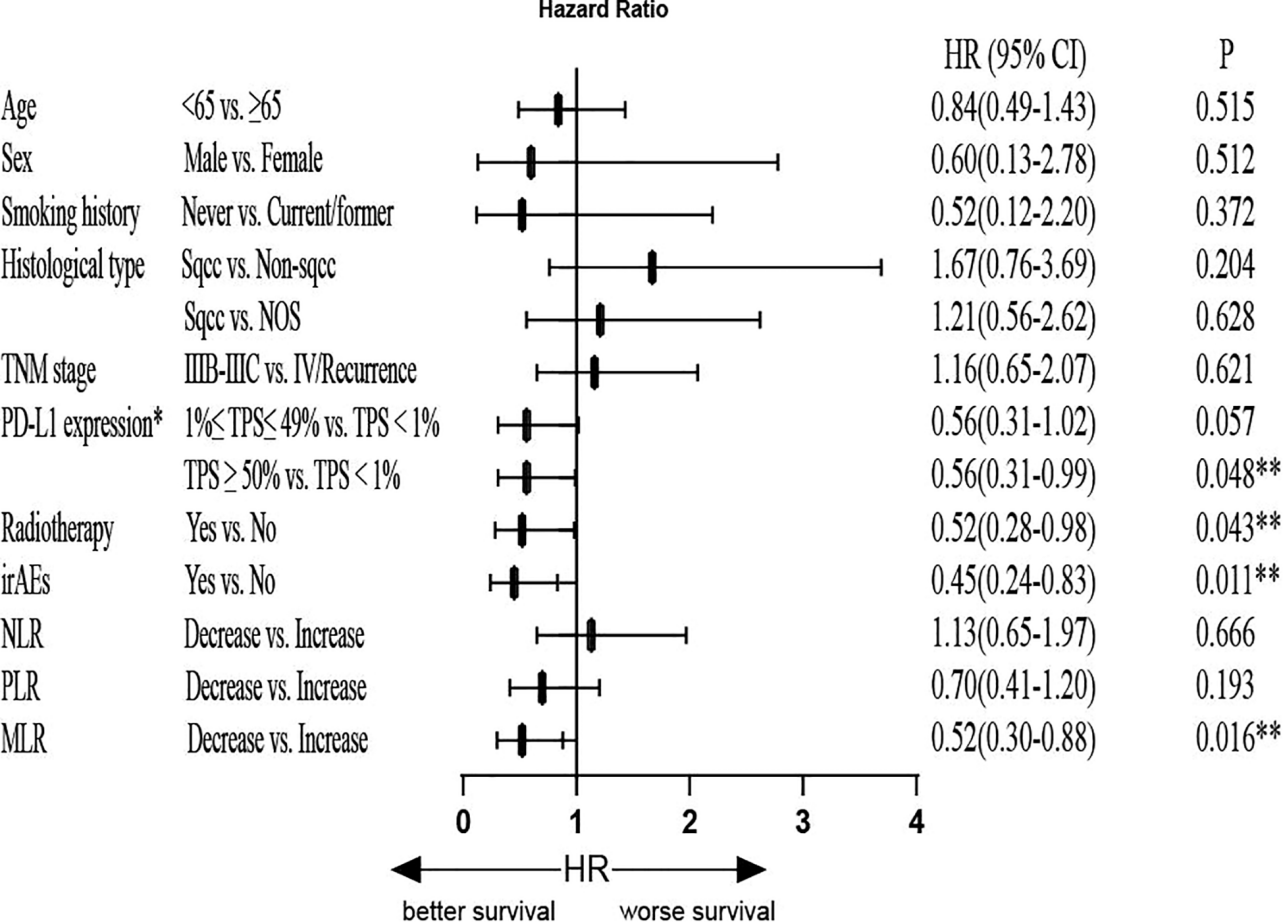
Multivariate Cox regression analysis of PFS. *Only for patients with available PD-L1 expression data (patients with unknown PD-L1 expression were excluded). PFS, progression-free survival; HR, hazard ratio; PD-L1: programmed cell death-Ligand 1; TPS, tumor proportion score; irAEs, immune-related adverse events; NLR, neutrophil-to-lymphocyte ratio; PLR, platelet-to-lymphocyte ratio; MLR, monocyte-to-lymphocyte ratio; **P<0.05 indicates statistical significance.

In the univariate analysis for PFS, no significant differences were detected with respect to patient age, sex, smoking history, histological type, TNM stage, and irAEs. However, the decreased MLR suggested longer PFS (HR=0.53, 95% CI, 0.33-0.84, P=0.007). The decreased NLR also suggested prolonged PFS (HR=0.63, 95% CI, 0.41–0.96, P=0.033). Next, we found that PD-L1 expression (TPS<1%) was associated with shorter PFS compared to both PD-L1 expression (1≤TPS ≤ 49%) (HR=0.50, 95% CI: 0.30–0.84, P=0.009) and PD-L1 expression (TPS≥50%) (HR=0.49, 95% CI: 0.27–0.88, P=0.017). Patients who received radiotherapy had prolonged PFS (HR=0.59, 95% CI: 0.35–0.99; P=0.045). To identify the independent predictors, Cox multivariate analysis was performed. In multivariate analyses, decreased MLR was significantly associated with prolonged PFS (HR=0.52, 95% CI: 0.30–0.88; P=0.016), and patients who had irAEs were significantly associated with prolonged PFS (HR=0.45, 95% CI: 0.24–0.83; P=0.011). In addition, patients who received radiotherapy were significantly associated with long longer PFS (HR=0.52, 95% CI: 0.28–0.98; P=0.043), and PD-L1 expression (TPS<1%) was associated with shorter PFS compared to PD-L1 expression (TPS≥50%) (HR=0.56, 95% CI: 0.31–0.99, P=0.048). NLR was not an independent predictive factor for PFS (P>0.05) (**Figure 5** and **Supplementary Figure 2**).

### Subgroup analysis of patients according to changes in MLR

All subgroup analyses showed that the decreased MLR group had significantly prolonged PFS compared to the increased MLR group, especially for subgroups of male, non-smokers, non-squamous, and ≥50% TPS (**Supplementary Figure 3A**). All subgroup analyses suggested high ORR for the decreased MLR group (**Supplementary Figure 3B**).

### Predictive value of changes in MLR, NLR, and PLR was superior to baseline MLR, NLR, PLR

The ROC curves were constructed using the baseline MLR, NLR, and PLR or changes in MLR, NLR, and PLR as the test variables. Specifically, we used different PFS time points (1, 2, and 3 years) and ORR as the state variable to plot time-dependent ROC curves. The area under the curve (AUC) represents the discriminative power of the test, and the closer the AUC is to 1.0, the higher the authenticity of the detection method. Then, the confidence interval (CI) for the AUC was calculated.

The results are shown in **Supplementary Figures 4**, **5**. The ROC curves showed that the AUC of changes in MLR were significantly larger than baseline MLR (AUC at 3 years: 0.813>0.152, P<0.001) or had a larger trend (AUC at 2 years: 0.661>0.412, P=0.071). The AUC of changes of NLR was larger than the baseline NLR (AUC at 2 years: 0.690>0.316, P<0.001; AUC at 3 years: 0.657>0.107, P<0.001). The AUC of changes in PLR was also larger than the baseline PLR (AUC at 2 years: 0.598>0.286, P<0.001). Therefore, we speculated that the dynamic changes of MLR, NLR, and PLR were superior to baseline MLR, NLR, and PLR for accurately predicting the long-term efficacy of first-line ICIs plus chemotherapy.

## Discussion

In this study, we showed that the decreased MLR was significantly associated with high ORR and long PFS in patients with stage IIIB-IV NSCLC treated with first-line PD-1 inhibitor combined with chemotherapy. The decreased NLR and PLR were also related to improved treatment outcomes (PFS or ORR). This finding suggested that altered MLR might be a predictive biomarker for patients with stage IIIB-IV NSCLC treated with first-line PD-1 inhibitor combined with chemotherapy. The predictive role of changes in MLR was effectuated regardless of the PD-L1 expression level. The changes in NLR and PLR might also have a specific predictive value and could be a supplement to predict prognosis.

The current results of this study could be explained by the following reasons. Firstly, monocytes can promote tumor progression and are recruited to primary or metastatic tumors, differentiating to tumor-associated macrophages (TAMs) (20). Secondly, lymphocytes are crucial in the host immune response and have potent anticancer activities that inhibit tumor cell proliferation and metastasis. Furthermore, it is speculated that increased lymphocyte levels are associated with improved clinical outcomes for various cancer types (21, 22). These studies supported our finding that the decreased MLR is associated with satisfactory treatment outcomes.

Furthermore, we observed that changes in MLR are associated with the treatment outcomes in advanced NSCLC patients treated with immunotherapy plus chemotherapy. Although previous studies have discussed the value of inflammatory markers in immunotherapy, only a few have mentioned their role in immunotherapy combined with chemotherapy. According to the guidelines, immunotherapy combined with chemotherapy has gradually become the most common treatment in advanced NSCLC patients without sensitizing driver mutations. Therefore, investigating the role of MLR in combination therapy was crucial. In addition, the inflammatory markers were usually investigated by identifying a specific cutoff. However, it is difficult to determine the cutoff value, and the correlation between changes in inflammatory markers and treatment outcomes was non-linear (15). The current study showed that changes in inflammatory markers were better than baseline inflammatory markers for longer-term efficacy prediction. Therefore, continuous monitoring of changes in these inflammatory markers during treatment might be a more reasonable method than identifying a cutoff for those patients who can benefit from immunotherapy combined with chemotherapy for a long time.

To the best of our knowledge, the expression of PD-L1 is an imperfect predictive marker, as even patients with positive PD-L1 may not benefit from PD-1 inhibitors. However, for patients with negative PD-L1 expression, PD-1 inhibitors might exhibit a satisfactory therapeutic effect. Therefore, we conducted a subgroup analysis of PFS and ORR according to the PD-L1 expression (TPS<1% *vs*. 1%≤TPS ≤ 49% *vs*. TPS≥50%) and found that decreased MLR was significantly associated with prolonged PFS in patients with PD-L1 expression. In addition, all subgroup analyses with PD-L1 expressions suggested high ORR for the decreased MLR group, especially the 1%≤TPS ≤ 49% and TPS<1% subgroup. These results suggested that changes in MLR might have a predictive value in all PD-L1-expressing populations, while the predictive value of PD-L1 in patients with negative expression is not effective. Therefore, changes in MLR might have a more effective predictive value than PD-L1 expression in patients with TPS<1%. Thus, we could assess the efficacy of PD-1 inhibitors combined with chemotherapy in different populations by observing the changes in MLR and PD-L1 expression, as inflammatory markers are readily available.

Nevertheless, the present study had some limitations. Firstly, this was a retrospective analysis with manual data extraction and entry, which could cause selection bias or introduce confounding factors. Nonetheless, several covariates reflected the disease characteristics and treatment effects that might interfere with the current analysis. Secondly, this study was performed in a single medical center, and only 140 patients were included, thereby limiting the generalizability of the results and necessitating large prospective studies in the future. However, our data maturity was high, and most patients were continuously followed up with progression events. Finally, due to insufficient observation time, we could not collect the mature data of OS, but blood indicators could be monitored dynamically, and subsequent data could be obtained easily.

In conclusion, decreased MLR was associated with improved treatment outcomes in patients with stage IIIB-IV NSCLC treated with first-line PD-1 inhibitor combined with chemotherapy. In addition, changes in MLR might have a predictive value in all the PD-L1-expressing populations. Decreased NLR and PLR also had a trend of enhanced survival, and changes in NLR and PLR might be complementary in predicting prognosis.

## Data availability statement

The datasets presented in this article are not readily available because Our data came from unfinished topics. Requests to access the datasets should be directed to zxychest0109@163.com.

## Ethics statement

The studies involving human participants were reviewed and approved by Shanghai Chest Hospital (Shanghai, China). The patients/participants provided their written informed consent to participate in this study. Written informed consent was obtained from the individual(s) for the publication of any potentially identifiable images or data included in this article.

## Author contributions

LZ and AX: Investigation, Writing - original draft, Writing - review and editing and Formal analysis. SW, JX: Data curation. YS, RZ: Visualization. JL, TC, WZ: Investigation and Resources. YL, XiZ: Project administration. BH, HZ: Supervision. WN: Investigation, Resources, Writing - review and editing and Conceptualization. XuZ: Writing-review and editing, Conceptualization and Formal analysis. All authors contributed to the article and approved the submitted version.
