# Supplementary material for: Decreased monocyte-to-lymphocyte ratio was associated with satisfied outcomes of first-line PD-1 inhibitors plus chemotherapy in stage IIIB-IV non-small cell lung cancer
Source: Front Immunol. 2023 Jan 26;14:1094378. doi: 10.3389/fimmu.2023.1094378 (PMC9909005; doi:10.3389/fimmu.2023.1094378)
Supplement: Supplementary file 1 [file DataSheet_1.docx]

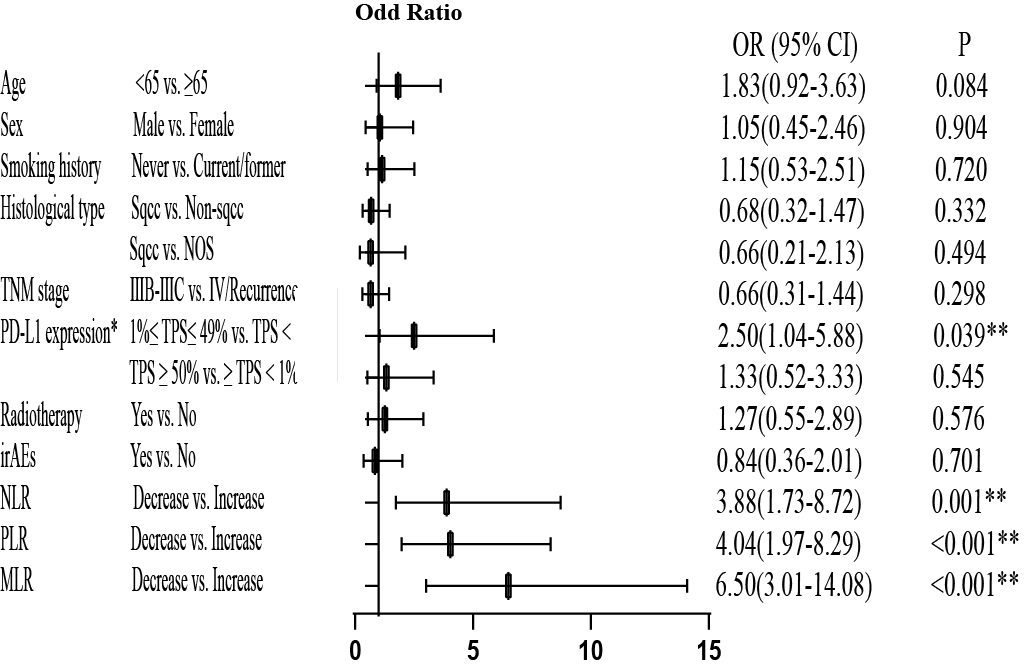


Supplementary Figure 1. Univariate analysis of ORR.

*Only for patients with available PD-L1 expression data (patients with unknown PD-L1 expression were excluded).

Abbreviations: ORR: objective response rate; OR, odds ratio; PD-L1: programmed cell death-Ligand 1; TPS, tumor proportion score; irAEs, immune-related adverse events; NLR, neutrophil-to-lymphocyte ratio; PLR, platelet-to-lymphocyte ratio; MLR, monocyte/lymphocyte ratio; ********, P<0.05 indicates statistical significance.


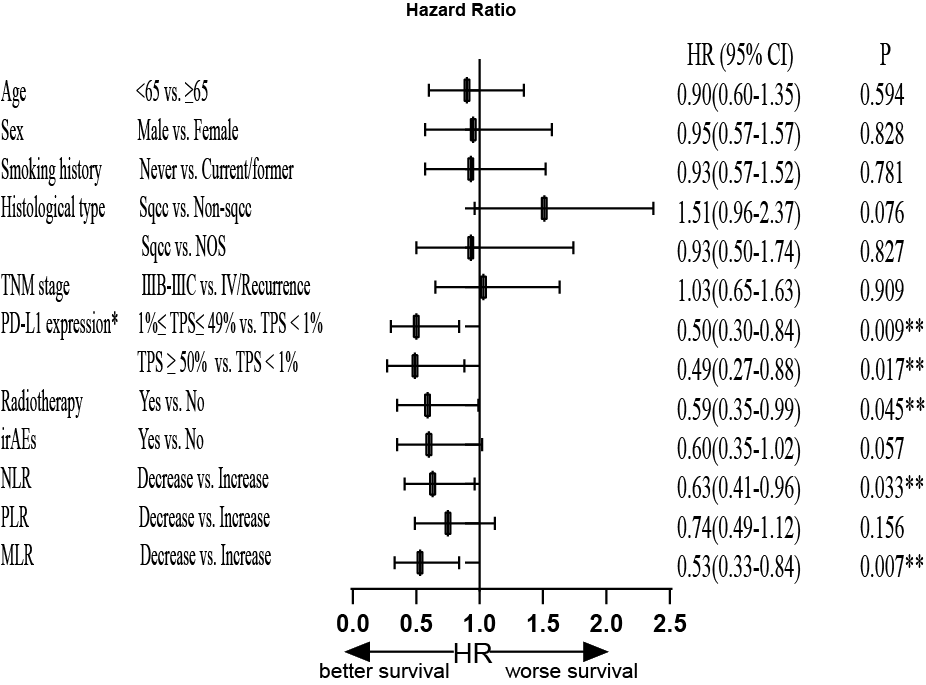


Supplementary Figure 2. Univariate Cox regression analysis of PFS.

*Only for patients with available PD-L1 expression data (patients with unknown PD-L1 expression were excluded).

Abbreviations: PFS, progression-free survival; HR, hazard ratio; PD-L1: programmed cell death-ligand 1; TPS, tumor proportion score; irAEs, immune-related adverse events; NLR, neutrophil-to-lymphocyte ratio; PLR, platelet-to-lymphocyte ratio; MLR, monocyte-to-lymphocyte ratio; ********, P<0.05 indicates statistical significance.


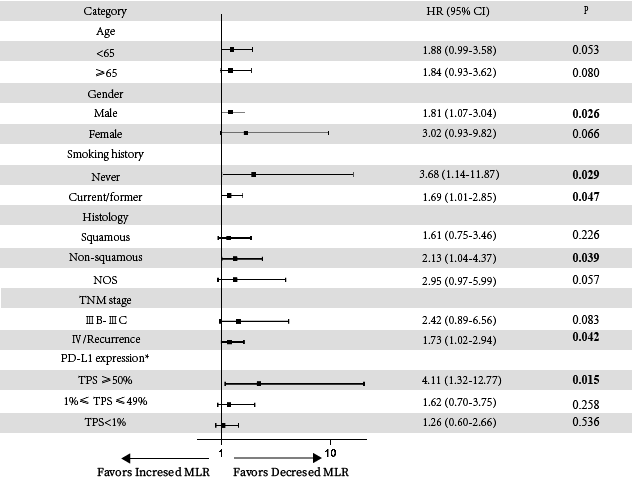


**A**

**
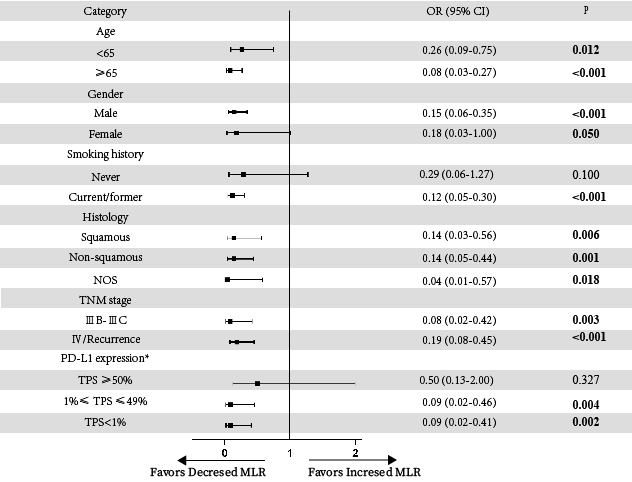
**

**B**

Supplementary Figure 3. Subgroup analysis of patients according to changes in monocyte-to-lymphocyte ratio (MLR).

1. Progression-free survival (PFS).
2. Objective response rate (ORR).

*Only for patients with available PD-L1 expression data (patients with unknown PD-L1 expression were excluded).

Abbreviations: NOS, not otherwise specified; PD-L1: programmed cell death-ligand 1; TPS, tumor proportion score; irAEs; immune-related adverse events.


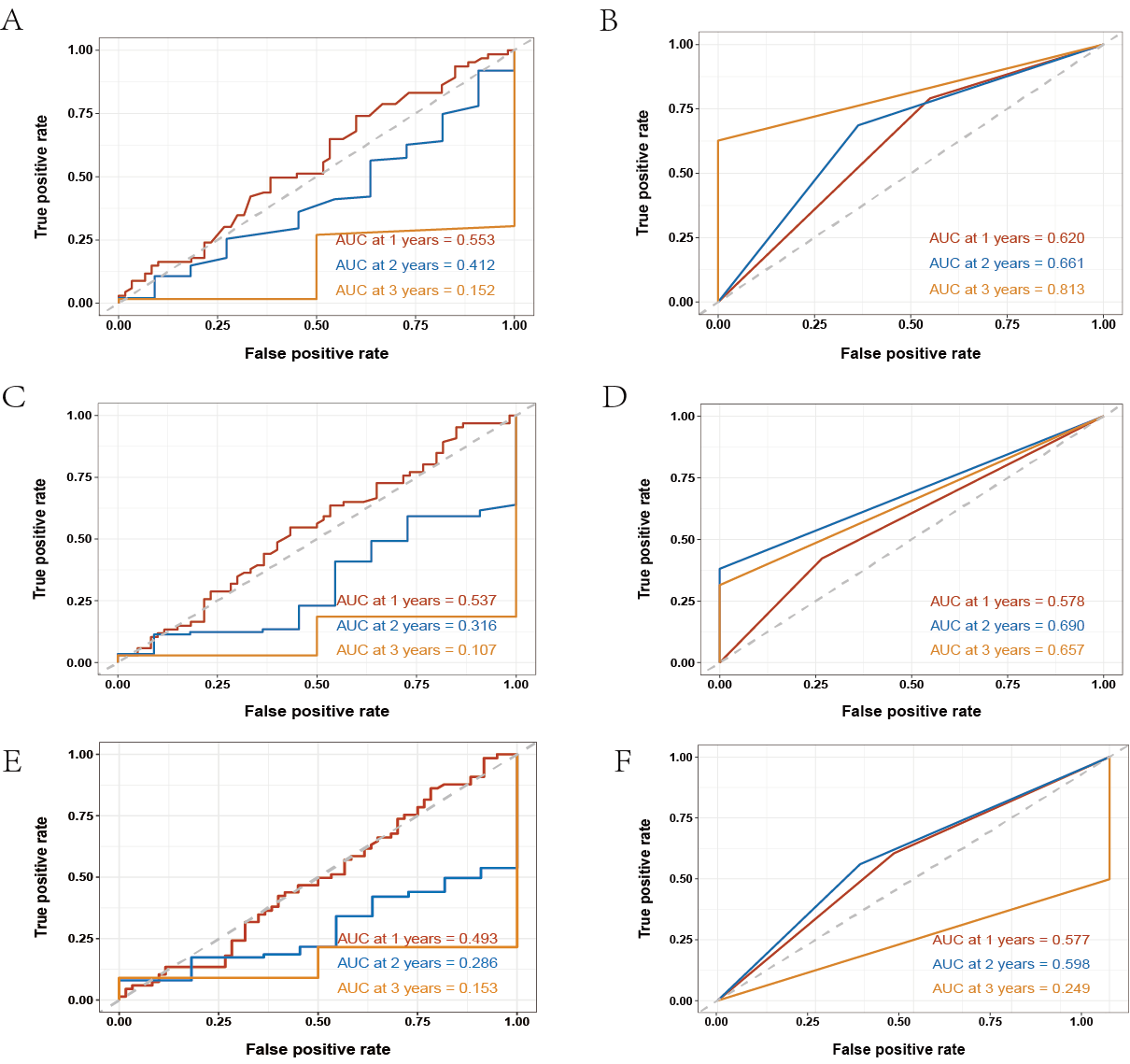


Supplementary Figure 4. Receiver operating characteristic (ROC) curves in A) baseline MLR; B) changes of MLR; C) baseline NLR; D) changes of NLR; E) baseline PLR; B) changes of PLR according to different PFS.


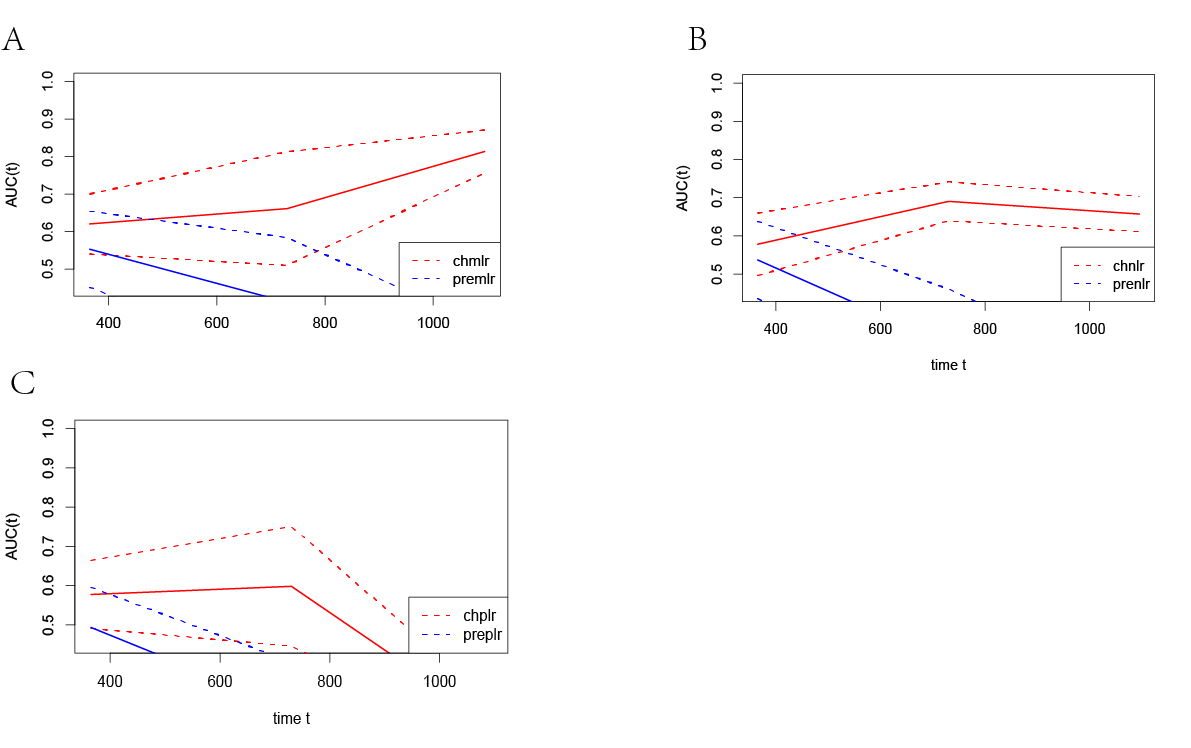


Supplementary Figure 5. Time-dependent ROC curves of A) changes of MLR and baseline MLR; B) changes of NLR and baseline NLR; C) changes of PLR and baseline PLR.

Abbreviations: chmlr: changes of MLR; premlr: baseline MLR; chnlr: changes of NLR; premlr: baseline NLR; chplr: changes of PLR; preplr: baseline PLR.
